# Design and Characterization of Durable Glass Fibre (GF)-Reinforced PLA and PEEK Biomaterials

**DOI:** 10.3390/polym17182536

**Published:** 2025-09-19

**Authors:** Asit Kumar Gain, Liangchi Zhang

**Affiliations:** 1 Shenzhen Key Laboratory of Cross-Scale Manufacturing Mechanics, Shenzhen 518055, China; a.gain@sustech.edu.cn; 2 SUSTech Institute for Manufacturing Innovation, Shenzhen 518055, China; 3 Department of Mechanics and Aerospace Engineering, Southern University of Science and Technology, Shenzhen 518055, China

**Keywords:** additive manufacturing, PEEK composite, mechanical properties, tribological performance

## Abstract

Poly(lactic acid) (PLA) and poly(ether-ether ketone) (PEEK) are widely recognized for their biocompatibility and processability in orthopaedic applications. However, PLA suffers from brittleness and limited thermal and mechanical stability, while PEEK, despite its better strength, does not fully replicate the mechanical and tribological performance of natural bone. This study explores the enhancement of structural and tribological properties in PLA- and PEEK-based composites reinforced with short glass fibres (S-GF) via additive manufacturing. Microstructural analysis confirms uniform GF dispersion within both polymer matrices, with no evidence of agglomeration, fibre pull-out, or interfacial debonding, suggesting strong fibre–matrix adhesion. The incorporation of GF significantly improved mechanical performance: microhardness increased by 38.3% in PLA and 36.3% in PEEK composites, while tensile strength increased by 25.1% and 13.4%, respectively, compared to plain polymers. These enhancements are attributed to effective stress transfer enabled by uniform fibre distribution and strong interfacial bonding. Tribological tests further demonstrate enhanced wear resistance, reduce damage propagation, and improved surface integrity under micro-scratching. These findings highlight the potential of GF-reinforced PLA and PEEK composites as high-performance materials for load-bearing biomedical applications, offering a balanced combination of mechanical strength and wear resistance aligned with the functional requirements of bioimplants.

## 1. Introduction

The development of durable and biocompatible materials for bioimplants remains a significant challenge in materials science and biomedical engineering. Historically, metallic biomaterials such as titanium and its alloys (e.g., Ti6Al4V), stainless steels, and cobalt–chromium (CoCr) alloys have dominated load-bearing implant applications owing to their better mechanical strength, fatigue resistance, corrosion resistance, and well-established clinical performance [1,2,3]. Among these, Ti6Al4V is notable for its combination of high specific strength, excellent biocompatibility and a relatively low elastic modulus compared to other metals, which helps to mitigate stress shielding effects and promotes osseointegration [4,5]. Nonetheless, metallic implants face inherent limitations including high density, radiopacity that complicates post-implantation imaging, susceptibility to ion release and corrosion under physiological conditions, and manufacturing challenges in producing patient-specific geometries without extensive machining [6,7]. Furthermore, the mismatch between the elastic modulus of artificial metallic implants and natural bone can lead to bone resorption and implant loosening over prolonged service periods [8].

In response to the aforementioned limitations associated with metallic implants, polymer-based biomaterials have emerged as promising alternatives in the development of next-generation biomedical devices. Among high-performance thermoplastics, PLA and PEEK have garnered considerable attention due to their favorable mechanical tunability, reduced density, and radiolucency—an attribute that facilitates enhanced postoperative imaging and long-term clinical monitoring [9,10]. PLA, a biodegradable polyester derived from renewable resources, has been widely employed in the fabrication of resorbable fixation devices and scaffolds for tissue engineering owing to its favorable biocompatibility and bioresorbable [11]. Nevertheless, its relatively low tensile strength, limited wear resistance and modest thermal stability restrict its use in load-bearing or long-term implant scenarios [12,13]. Conversely, PEEK, a semi-crystalline high-performance polymer [14], exhibits better tensile strength and elastic modulus compared to other biomedical polymers, coupled with exceptional chemical inertness and thermal stability up to 343 °C. These attributes render PEEK highly suitable for permanent implant applications, including spinal cages, dental implants and joint replacement components [15,16,17]. Nevertheless, despite these favorable properties, pure PEEK often exhibits inadequate tribological performance under articulating or load-bearing conditions, thereby necessitating the incorporation of reinforcing agents to enhance its wear resistance and long-term mechanical durability.

To address these intrinsic limitations, substantial research efforts have been dedicated to the development of PEEK-based composites reinforced with a variety of ceramic and fibre-based particulates. These reinforcements are intended to enhance the mechanical, thermal, and tribological characteristics of the base polymer, thereby broadening its applicability to complex, load-bearing bioimplants. Conventional fabrication techniques, including powder metallurgy-based extrusion, compression molding and injection molding, have been employed to achieve a uniform dispersion of reinforcements within the matrix, thereby enhancing the mechanical properties [18,19,20]. Common reinforcements include hydroxyapatite (HAp), alumina (Al_2_O_3_), Silica (SiO_2_), carbon fibre (CF) and glass fibres (GF)-each contributing to improvements in stiffness, hardness and wear resistance, while preserving biocompatibility and bio-functionality [18,21,22,23]. For instance, Deng et al. developed PEEK composites reinforced with nano-HAp/CF, which significantly improved mechanical properties, and in vivo tests showed greater bone formation compared to pure PEEK, confirmed by 3D micro-CT and 2D histology [18].

Among the various reinforcement strategies, the incorporation of S-GFs into PEEK has been extensively validated as an effective approach to enhance both mechanical and tribological performance. The addition of 0–50 wt% S-GF, fabricated via injection molding, results in significant improvements in flexural modulus, compressive strength and Vickers hardness [24]. The fibre–matrix interfacial adhesion is widely recognized as a critical determinant of composite performance, as it governs the effectiveness of stress transmission, interfacial energy dissipation, and overall damage tolerance under cyclic loading and contact sliding conditions of paramount relevance to load-bearing biomedical applications [25]. Within the context of fused deposition modeling (FDM), recently, a widely used additive manufacturing (AM) technique, the orientation and distribution of short fibres can be modulated through careful control of processing parameters, including nozzle temperature, print speed, layer height and raster orientation. This enables the production of components with architecturally anisotropic mechanical and tribological properties tailored to match physiological load paths and anatomical complexity [26,27]. Moreover, FDM can also be employed to fabricate porous PEEK scaffolds with bone-mimicking structures [28]. These features improve compatibility with the body, support bone growth, and reduce stress shielding [29,30]. Despite the demonstrated potential of fibre-reinforced polymer systems fabricated via AM, comprehensive investigations that elucidate the complex interdependence between microstructural attributes—such as fibre orientation distribution, dispersion homogeneity, interfacial integrity, and matrix crystallinity—and the resultant mechanical performance under physiologically relevant conditions remain scarce. This represents a critical knowledge gap, as biomedical implants are routinely subjected to multifaceted loading environments comprising dynamic compression, tension, and reciprocating slides. The synergistic effects of these stressors on wear behavior, and structural longevity must be systematically evaluated to inform the rational design of next-generation polymer composite implants with enhanced clinical reliability and long-term functional performance [31].

Therefore, the present study undertakes a systematic and comprehensive investigation into the structural design, mechanical response, and tribological behavior of short glass fibre (S-GF)-reinforced PLA and PEEK composites fabricated via FDM. This research aims to critically assess the viability of these reinforced polymer systems as lightweight, corrosion-resistant, and wear-tolerant alternatives for next-generation biomedical implants. The specific objectives of this work are fourfold: (i) to fabricate GF-reinforced PLA and PEEK composites; (ii) to characterize their mechanical properties and failure behavior; (iii) to assess their tribological performance to elucidate the dominant wear mechanisms; and (iv) to perform detailed microstructural analyses to establish correlations between structural features and the resulting mechanical and tribological responses.

## 2. Materials and Methods

### 2.1. Manufacturing PLA- and PEEK-Based Composition

**Figure 1:**
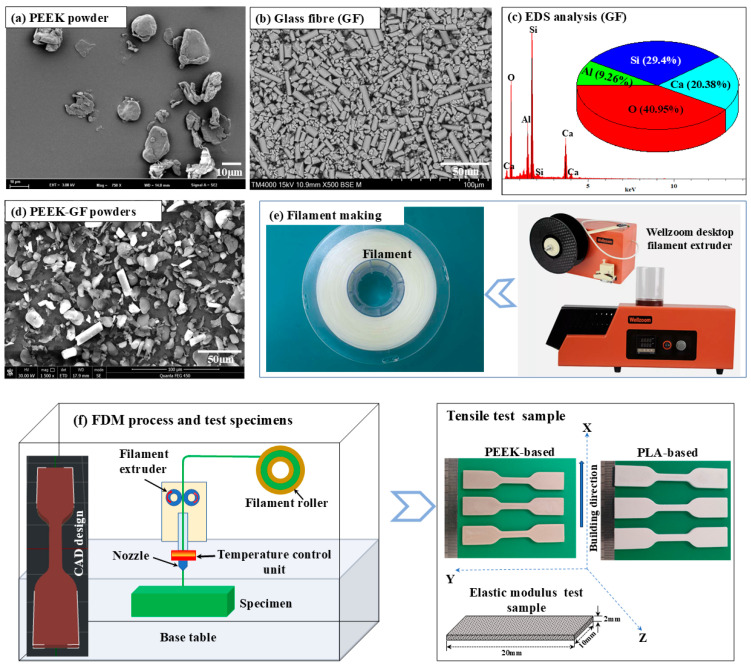
Microstructural characterization of the raw materials and schematic representation of the experimental procedure: (**a**) SEM micrograph of PEEK powder; (**b**,**c**) SEM micrograph and corresponding EDS analysis of GF; (**d**) SEM micrograph of the blended powder mixture; (**e**) schematic illustration of the filament fabrication process; and (**f**) schematic diagram of the additive manufacturing technique utilized for PLA- and PEEK-based composites, alongside the configuration of the test specimens.

The fabrication of glass fibre (GF)-reinforced PLA and PEEK biomaterials was achieved using fused deposition modeling (FDM), beginning with the compounding of 5 wt.% GF (Nanjing Joyfulchem Co., Ltd., Nanjing, China) into commercial-grade PLA pellets and PEEK powders (Strem Chemical, Inc., Newburyport, MA, USA). The PEEK powders exhibited a pellet-like irregular morphology with an average dimension of approximately 20 µm, as shown in Figure 1a. The GF reinforcement displayed a cylindrical shape with an average diameter of ~7 µm and lengths ranging between 10 and 125 µm as shown in Figure 1b, primarily composed of silicon (Si), calcium (Ca), aluminum (Al), and oxygen (O) elements confirmed by EDS profile Figure 1c. These powders were mixed using low-energy ball milling at a rotational speed of 15 rpm for 24 h to achieve uniform dispersion of the GF within the polymer matrix employing ZrO_2_ balls (5 mm in diameter) as the milling media. The mixed powders were characterized by SEM, as shown in Figure 1d, confirming that the glass fibers (GF) were uniformly dispersed within the PEEK matrix. The homogenized mixture was then processed through a single-screw extruder to ensure uniform fibre distribution. The resulting materials were subsequently extruded into continuous filaments with a diameter of 1.75 mm using a single-screw extruder (Wellzoom desktop filament extruder), with extrusion temperatures controlled at 190–210 °C for PLA composites and 360–400 °C for PEEK composites as shown in Figure 1e. The extruded filaments were then dried in an oven at 70 °C for 24 h to eliminate residual moisture prior to FDM printing. These dried filaments were printed using an FDM 3D printer (IEMAI MAGIC-HT-M) equipped with a steel nozzle to accommodate the abrasive nature of the GF, as illustrated in Figure 1f. Printing parameters were optimized for each material system, with nozzle temperatures of 210 °C for PLA and 390 °C for PEEK, bed temperatures set at 60 °C and 90 °C, respectively, layer heights ranging from 0.1 to 0.2 mm, and a rectilinear infill pattern at 100% density to ensure structural integrity and maximize mechanical performance. To enhance interlayer adhesion and reduce residual stress, printing was conducted within a heated enclosure that maintained elevated ambient temperatures. Post-processing involved annealing the printed specimens at 80 °C for 2 h (PLA composites) and at 200 °C for 4 h (PEEK composites) to improve crystallinity and relieve internal stresses, thereby enhancing durability. Finally, the printed components were machined and finished to precise dimensions for subsequent mechanical testing and microstructural evaluation.

### 2.2. Characterization of Microstructure and Mechanical Property

For microstructural and mechanical characterization, the printed specimens were polished by standard metallographic technique using an automated polishing apparatus (FORCIPOL 102, Metkon, Mauldin, SC, USA). Initial surface was grinded with silicon carbide (SiC) abrasive papers of progressively finer grit size, ranging from P800 to P4000. This was followed by final polishing using a colloidal silica suspension containing 40 nm particles (Goral A90004, 0.04CR) to attain a nanoscale surface finish suitable for high-resolution imaging and micro-scratch testing. A representative subset of the polished specimens was used for microstructural analysis to examine the morphology and spatial distribution of GF within the polymer matrix using field emission scanning electron microscopy (FE-SEM, ZEISS Merlin, Oxford, UK), equipped with a Schottky-type thermal field emission electron source. To suppress surface charging effects and enhance imaging fidelity, specimens were sputter-coated with an approximately 5 nm thick platinum (Pt) layer under high-vacuum conditions (1 × 10^−6^ mbar) using a precision sputter coater (Q150V Plus, Quorum Technologies Ltd., Lewes, UK). In parallel, microhardness mapping was conducted on a separate subset of specimens employing a DuraScan EMCO Vickers hardness tester (Model 70-G5, GmbH, Kellau, Austria). A regular 3 × 3 indentation matrix was applied across the polished surface under a constant test load of 0.2 kgf and a dwell time of 10 s per indent, enabling spatially resolved quantification of hardness variations associated with microstructural heterogeneity.

For elastic modulus measurements, an IMCE RFDA LT80 (IMCE, NV Slingerweg, Belgium) system was used to characterize the PEEK-based nanocomposites. Rectangular specimens (15 mm × 8 mm × 2 mm) were suspended on a thin metal wire inside a temperature-controlled chamber, and each condition repeated 5 times. At room temperature, a thin Al_2_O_3_ bar impacted the specimen to induce excitation, and the resulting vibrational response was recorded with a microphone. The elastic modulus was then calculated from the flexural resonance frequency following ASTM standards [32].

Additionally, to evaluate the tensile properties, standard tensile specimens of PLA- and PEEK-based materials were fabricated via 3D printing according to the dimensions shown in Figure 1f. Tensile tests were performed at room temperature using a universal testing machine (INSTRON 68FM-100, Norwood, MA, USA) under a constant strain rate of 0.001 s^−1^.

### 2.3. Evaluation of Tribological Performance

The tribological performance of the PLA- and PEEK-based composites was evaluated through micro-scratch testing using a high-precision multi-functional tribometer integrated with a 3D optical profilometer (MFT-5000, Rtec Instruments, San Jose, CA, USA). The tests were conducted using a spherical diamond indenter repeated three times under a controlled range of normal loads (15–30 N), applied at a constant sliding velocity of 0.2 m/s over a sliding distance of 1.5 mm at room temperature. After micro-scratching, the worn surfaces were examined using OM and SEM techniques to elucidate the underlying wear mechanisms.

## 3. Results and Discussion

### 3.1. Microstructural Characterization

**Figure 2:**
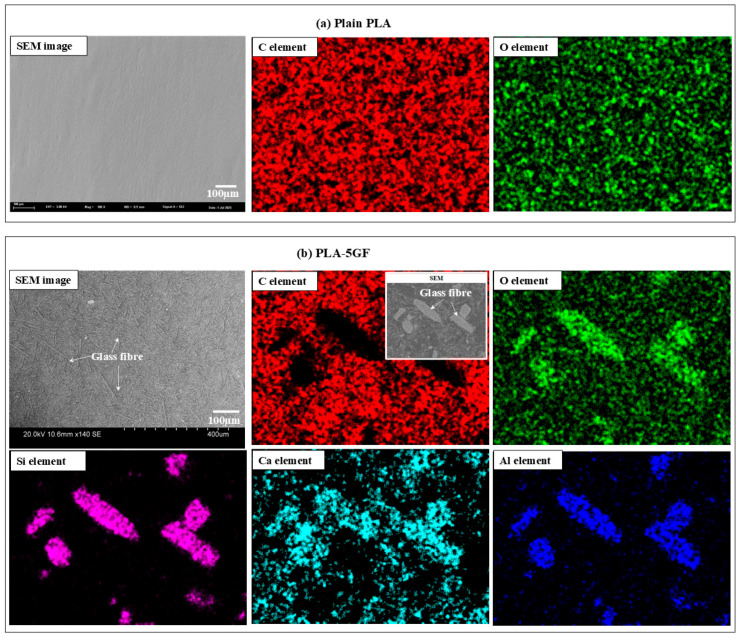
Microstructural characterization of PLA-based composites: (**a**) SEM micrograph accompanied by EDS mapping and corresponding elemental analysis of the pure PLA; (**b**) SEM micrograph, EDS mapping, and elemental analysis of the PLA-5GF composite, illustrating the dispersion of GF and elemental distribution within the polymer matrix.

Figure 2 presents a detailed microstructural evaluation that elucidates the morphological and compositional characteristics of the PLA-based composites. As shown in Figure 2a, the SEM micrograph of the pure PLA revealed a relatively smooth and homogeneous surface. The corresponding EDS elemental mapping predominantly exhibited peaks for carbon (C) and oxygen (O), aligning with the expected elemental composition of pure PLA matrix. Conversely, Figure 2b illustrates the microstructure of the PLA composite reinforced with 5GF, where the presence of embedded fibrous structures was clearly evident, confirming the successful incorporation of the reinforcement phase into the polymer matrix. The EDS mapping of this composite indicated a heterogeneous distribution of elements, with prominent signals corresponding to silicon (Si), aluminum (Al), oxygen (O) and calcium (Ca)—key constituents of the glass fibre—thereby verifying their uniform dispersion within the polymer matrix. These observations confirmed the structural integrity of the composite and demonstrated the efficacy of the fabrication process.

**Figure 3:**
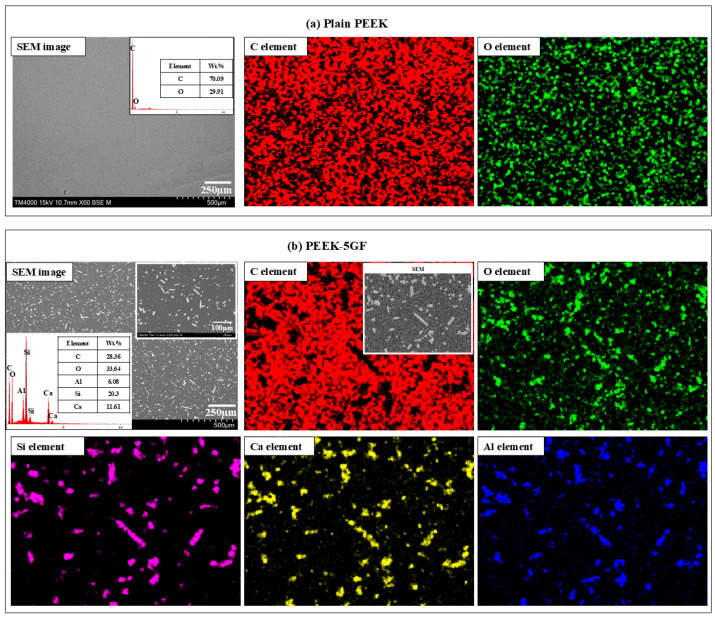
Microstructural characterization of PEEK-based composites: (**a**) SEM micrograph accompanied by EDS mapping and corresponding elemental analysis of the pure PEEK; (**b**) SEM micrograph, EDS mapping, and elemental analysis of the PEEK-5GF composite, illustrating the dispersion of GF and elemental distribution within the polymer matrix.

Figure 3 illustrates the microstructural characterization of the PEEK-based composites through SEM coupled with EDS mapping and quantitative elemental analysis. This integrated approach enabled to elucidate the morphology and elemental composition of both the pure and PEEK-based composites, thereby providing critical insights into the material architecture and phase homogeneity. In Figure 3a, the SEM micrograph of the pure PEEK sample revealed a topographically smooth and featureless surface with no observable microstructural discontinuities, inclusions, or phase separations. This uniform morphology is indicative of the high degree of structural homogeneity typical of thermoplastic polymers processed under controlled conditions. The corresponding EDS elemental mapping exhibited a consistent and spatially uniform distribution of carbon (C) and oxygen (O), in full agreement with the molecular structure of PEEK-based polymer. In contrast, Figure 3b presents the SEM micrograph and EDS analysis of the PEEK composite reinforced with 5GF. The SEM micrograph distinctly revealed the presence of dispersed fibrous structures embedded within the polymer matrix. These GF exhibited a random orientation and were distributed relatively uniformly throughout the PEEK matrix, which is critical for achieving isotropic reinforcement behavior. Notably, the fibre–matrix interfacial regions exhibited intimate contact without visible signs of interfacial debonding, fibre pull-out, or matrix cracking, suggesting strong interfacial adhesion between the thermoplastic matrix and the inorganic reinforcement. Such interfacial integrity is critical in semi-crystalline polymer composites, where stress transfer across the interface governed the composite’s macroscopic performance. The EDS mapping further elucidated the phase morphology, revealing elementals with the GF phase—namely silicon (Si), oxygen (O), calcium (Ca), and aluminium (Al), localized specifically to the fibre regions. These elemental distributions are consistent with the composition of GF and confirmed their successful integration within the PEEK matrix. The microstructural observation coupled with EDS analyses provide compelling evidence of the successful incorporation and homogeneous dispersion of GF within the PEEK matrix, along with chemically and structurally stable fibre–matrix interfaces. These microstructural features are expected to significantly influence the composite’s mechanical response by enhancing stiffness, strength, and thermal dimensional stability—critical parameters for high-performance polymeric materials intended for structural and functional applications.

### 3.2. Mechanical Property Evaluation

**Figure 4:**
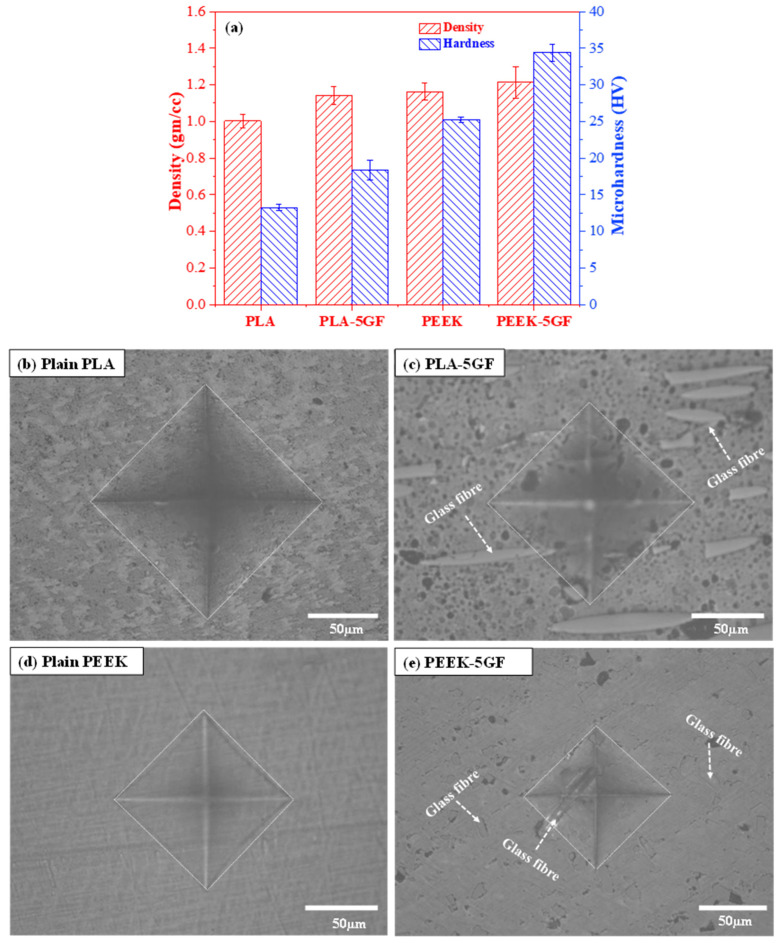
Microhardness characterization of PLA- and PEEK-based composites: (**a**) comparative analysis of relative density and microhardness values for PLA- and PEEK-based composites; representative optical micrographs of residual indentation imprints on the surfaces of (**b**) PLA, (**c**) PLA-5GF, (**d**) PEEK and (**e**) PEEK-5GF composites, respectively, illustrating the deformation response under applied load.

To comprehensively evaluate the influence of GF reinforcement on polymer matrices, the microhardness characteristics of both PLA- and PEEK-based composites were systematically characterized, and the results were presented in Figure 4. These results provide quantitative insights, complemented by qualitative microstructural evidence, into the localized deformation behavior under indentation. The density of all specimens varied from 1.0 to 1.21 gm/cc as shown in Figure 4a. However, the incorporation of GF into both thermoplastic matrices led to a pronounced enhancement in microhardness. Specifically, plain PLA exhibited a relatively low hardness of approximately 13.3 ± 0.46 HV, whereas its GF-reinforced counterpart (PLA-5GF) demonstrated a significantly increased value of 18.4 ± 1.38 HV. This notable improvement highlights the effectiveness of GF in restricting plastic deformation and enhancing the microscale load-bearing capability of the composite material. A more substantial improvement was observed in the PEEK-based composite, wherein the inherently higher hardness of plain PEEK was further elevated upon GF incorporation. The inherent microhardness of plain PEEK, measured at 25.5 ± 0.36 HV, increased markedly to 34.4 ±1.16 HV with the addition of GF. This enhancement can be primarily attributed to the efficient stress transfer between the stiff GF reinforcements and the semi-crystalline PEEK matrix, which effectively suppresses matrix yielding and facilitates greater resistance to localized indentation-induced plasticity.

Figure 4b–e provide representative optical micrographs of the residual indentation imprints on the surfaces of the tested specimens, offering insight into the material response to localized mechanical stress. The indentation imprint on plain PLA (Figure 4b) is characterized by a relatively large and deep impression, accompanied by visible plastic deformation zones surrounding the indent, indicating limited resistance to localized compressive stress. In contrast, the PLA-5GF composite (Figure 4c) displayed a noticeably smaller indentation area, indicative of enhanced surface stiffness and a more constrained plastic flow, resulting from the presence of rigid GF uniformly distributed within the PLA matrix. Similarly, the plain PEEK specimen (Figure 4d) showed a moderate indentation profile with less pronounced deformation zones compared to PLA, consistent with its better mechanical performance and higher intrinsic hardness. The PEEK-5GF composite (Figure 4e), however, showed the smallest and shallowest indentation imprint among all tested materials, reflecting a further increase in resistance to plastic deformation due to the synergistic interaction between the PEEK matrix and the embedded GF. The reduced depth and area of the residual imprints in the reinforced systems not only confirm the effectiveness of GF reinforcement but also suggest improved dimensional stability and durability under service conditions involving surface contact loading.

**Figure 5:**
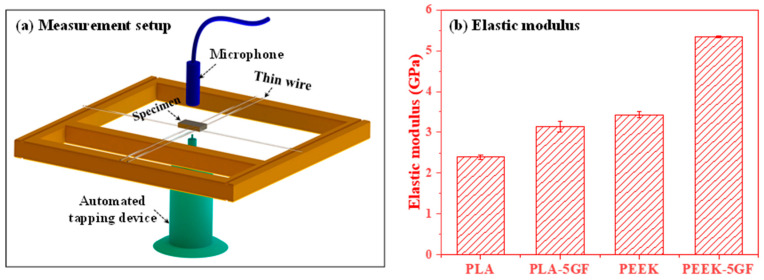
(**a**) Schematic representation of the experimental setup for measuring the elastic modulus using impulse technique and (**b**) the associated elastic modulus values of the PLA and PEEK-based composites, highlighting the effect of the GF reinforcement on the mechanical properties.

Figure 5 presents a comprehensive elucidation of both the experimental methodology and resultant data concerning the elastic modulus characterization of PLA- and PEEK-based composites. As shown in Figure 5a, the experimental setup is based on the impulse excitation technique (IET), a highly precise and non-destructive method employed to evaluate the elastic properties of solid materials. Figure 5b presents the measured elastic modulus values for both pure and GF-reinforced PLA and PEEK composites. The elastic modulus of pure PLA and PLA-GF was determined to be 2.39 GPa and 3.14 GPa, respectively, both falling below the reported range for human cortical bone (3–30 GPa) [33,34]. In contrast, pure PEEK and PEEK-GF exhibited elastic moduli of 3.43 GPa and 5.34 GPa, respectively, with the incorporation of glass fibers markedly enhancing the stiffness of PEEK-GF, thereby bringing its mechanical response within the range of cortical bone. The incorporation of GF in polymer matrix increased elastic modulus both polymer matrices, indicating its significant role in increasing the stiffness of the polymer matrices. This improvement can be primarily attributed to the high intrinsic rigidity of the GF and their ability to facilitate efficient load transfer across the fibre–matrix interface. These findings provide the critical role of GF reinforcement in modulating the elastic behavior of polymer composites and underline its strategic importance in the rational design of materials for advanced engineering applications necessitating elevated stiffness and dimensional stability.

**Figure 6:**
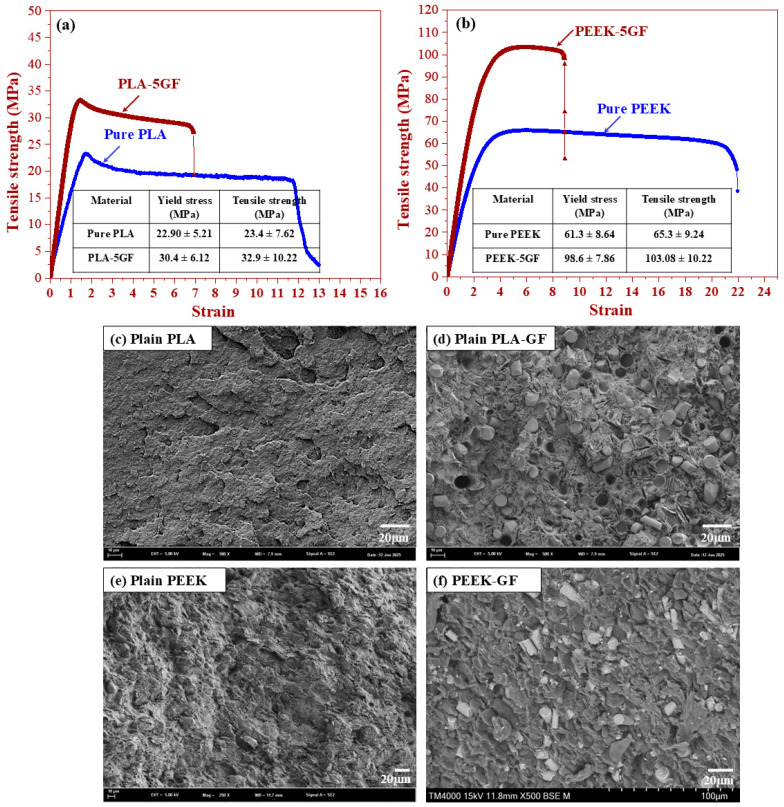
Mechanical properties and fracture surface characterization of polymer composites: (**a**) stress–strain curve of PLA-based composite; (**b**) stress–strain curve of PEEK-based composite; and SEM images of the fracture surfaces of (**c**) plain PLA, (**d**) PLA-5GF, (**e**) plain PEEK and (**f**) PEEK-5GF.

Figure 6 presents a comprehensive analysis of the mechanical behavior and fracture surface morphology of PLA- and PEEK-based polymer composites. The stress–strain curves of these composites are presented in Figure 6a and Figure 6b, respectively. For the PLA-based system (Figure 6a), the incorporation of GF yielded a pronounced improvement in tensile strength relative to the plain PLA matrix. The composite exhibited a steeper initial elastic regime and a higher ultimate tensile stress, underscoring the reinforcing efficacy of the stiff GF within the relatively ductile polymer matrix. These enhancements can be predominantly attributed to the efficient load transfer from the matrix to the embedded GF through interfacial adhesion and mechanical interlocking at the fibre–matrix interface. A comparable enhancement was observed in the PEEK-based system (Figure 6b), wherein the integration of GF significantly enhanced the overall tensile response. In comparison to pure PEEK, which exhibited a yield stress of 61.3 ± 8.64 MPa and a tensile strength of 65.3 ± 9.24 MPa, the PEEK-5GF composite demonstrated significantly enhanced values of 98.6 ± 7.86 MPa and 103.08 ± 10.22 MPa, respectively. Notably, these values fall within the typical range of ultimate tensile strength for cortical bone, reported to be approximately 50–150 MPa [18]. Such a pronounced enhancement can be ascribed to the effective redistribution of internal stresses, facilitated by the high intrinsic stiffness of the glass fibers and the strong interfacial adhesion between the fibers and the polymer matrix.

To further elucidate the underlying failure mechanisms, SEM observation was conducted on the fracture surfaces, and the results are presented in Figure 6c–f. The fracture surface of the plain PLA (Figure 6c) revealed relatively smooth and featureless regions, typical of brittle fracture with minimal plastic deformation. Conversely, the PLA-5GF composite (Figure 6d) exhibited a markedly rougher morphology characterized by fibre pull-out and fibre breakage, signifying the activation of energy-dissipative mechanisms such as fibre debonding, and interfacial sliding—indicative of enhanced fracture toughness in the reinforced system. Similarly, the fracture surface of pure PEEK (Figure 6e) demonstrated features consistent with ductile failure, including extensive plastic flow and shear deformation. However, upon reinforcement with GF, the PEEK-5GF composite (Figure 6f) displayed a significantly more complex fracture topography, incorporating fibre–matrix interfacial debonding, residual fibre protrusion, and pronounced matrix tearing. These microstructural features provided compelling evidence for a transition toward a more energy-absorptive failure regime, governed by synergistic toughening mechanisms such as fibre bridging, and frictional sliding at the interfacial region. The integrated analysis of mechanical performance and fractured surface morphology clearly demonstrated the substantial benefits of GF incorporation in enhancing the structural integrity, load-bearing capacity, and damage tolerance of both PLA- and PEEK-based polymer matrices.

### 3.3. Assessment of Tribological Performance

**Figure 7:**
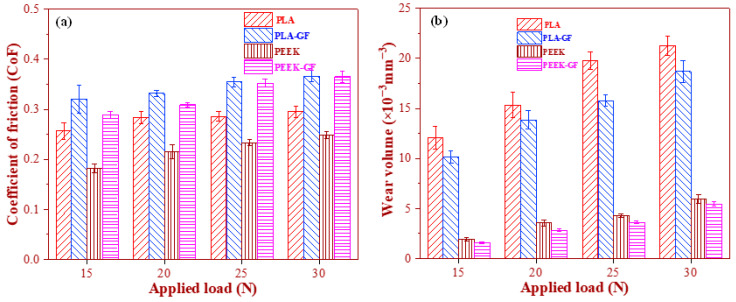
Comparative assessment of the tribological behavior of polymer composites conducted through micro-scratch testing. (**a**) Coefficient of friction (CoF) and (**b**) wear loss of PLA-based and PEEK-based composites as a function of the applied load.

Figure 7 provides a comparative analysis of the tribological behavior of PLA- and PEEK-based polymer composites, as evaluated using micro-scratch testing. Figure 7a presents the evolution of the coefficient of friction (CoF) for the PLA- and PEEK-based systems, respectively, while Figure 7b shows the corresponding volumetric wear loss profiles under identical testing conditions. The results revealed a marked divergence in tribological performance between the two polymer matrices, reflecting differences in their inherent mechanical properties, thermal resistance, and interfacial behavior under sliding contact. The CoF profiles for the PLA-based composites consistently exhibited higher frictional responses relative to the PEEK-based counterparts. This pronounced frictional behavior is primarily attributed to the lower thermal stability and reduced toughness of PLA. Under localized loading, the brittle nature of the PLA matrix facilitated increasing ploughing and adhesion at the sliding interface, thereby increasing resistance to sliding. The addition of GF further increased CoF in the PLA-based system. This increment raised from the protrusion of rigid GF reinforcements at the sliding interface, which enhanced micro-asperity contact and abrasive interactions, ultimately impeding lateral motion. Furthermore, fibre–matrix interfacial zones may act as localized stress concentrator, facilitating micro-fracture initiation and debris generation—both of which contribute to frictional instability and increased CoF values. Conversely, the PEEK-based composites exhibited better tribological performance, as evidenced by their lower and more stable CoF profiles across the tested load range. This behavior is attributed to the semi-crystalline structure, high thermal resistance, and inherent toughness of the PEEK matrix, collectively suppress adhesion and ploughing effects during sliding. Although the introduction of GF into the PEEK matrix results in a moderate increase in CoF, the overall frictional response remains stable. This stability is indicative of effective load transfer mechanisms and suppressed matrix deformation, thereby reflecting the favorable interfacial synergy between the GF reinforcement and the PEEK matrix.

Figure 7b further corroborate these findings by illustrating the wear loss characteristics of each system. The results clearly indicated that the PLA-based materials experience significantly greater material removal compared to their PEEK-based counterparts, thereby underscoring the inferior wear resistance of the PLA matrix. Quantitatively, the wear volume of plain PLA at nominal forces of 15 N and 30 N was measured to be approximately 12.09 ± 1.12 × 10^−3^ mm^3^ and 21.25 ± 0.94 × 10^−3^ mm^3^, respectively. In contrast, the incorporation of GF into the PLA matrix leads to reduce in wear loss, with PLA-5GF exhibiting wear volumes of 10.15 ± 0.64 × 10^−3^ mm^3^ at 15 N and 18.68 ± 1.09 × 10^−3^ m^3^ at 30 N. This improvement can be attributed to the reinforcing effect of GF, which enhanced the load-bearing capacity of the matrix and introduced a degree of mechanical interlocking that mitigates excessive material removal. Similarly, the PEEK-based composites demonstrated better wear performance. For plain PEEK, the wear volumes were approximately 1.94 ± 0.21 × 10^−3^ mm^3^ at 15 N and 5.96 ± 0.39 × 10^−3^ mm^3^ at 30 N. With the addition of GF, these values further decrease to 1.59 ± 0.064 × 10^−3^ mm^3^ and 5.46 ± 0.21 × 10^−3^ mm^3^, respectively, indicating that the GF reinforcement is similarly effective in enhancing the wear resistance of the PEEK matrix. Notably, the wear volume of PEEK-based composites remains an order of magnitude lower than that of PLA-based systems under all tested conditions, reflecting the intrinsic superiority of PEEK in tribological applications.

**Figure 8:**
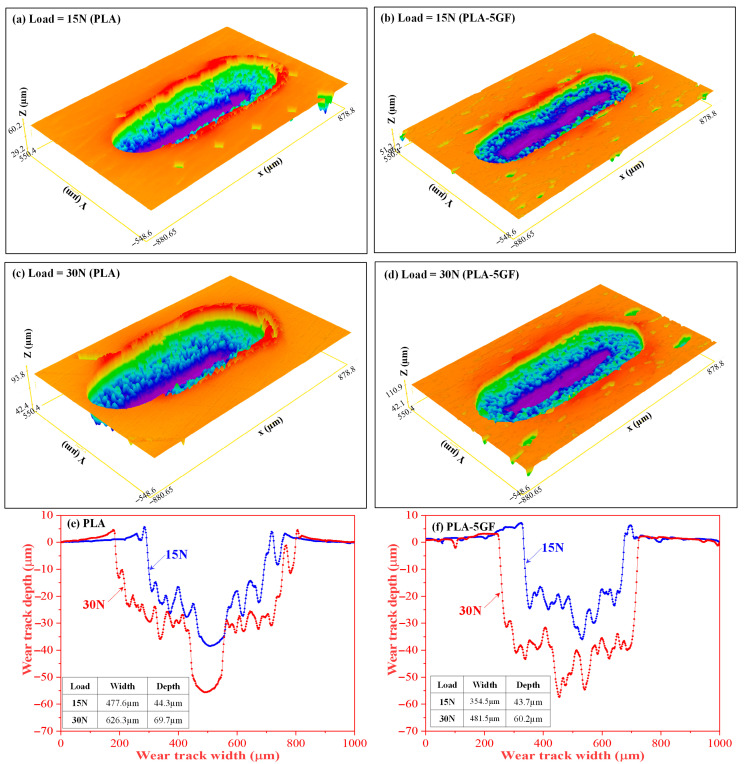
In situ optical microscopy (OM) images illustrating the micro-scratch morphologies of PLA-based composites: (**a**,**c**) plain PLA and (**b**,**d**) PLA-5GF, subjected to scratching under varying nominal loads. Images (**a**,**b**) correspond to a nominal load of 15 N, while (**c**,**d**) present the morphologies under a higher load of 30 N. The corresponding surface profiles are shown in (**e**,**f**), providing insight into the depth and deformation characteristics associated with each loading condition (blue color line for 15 N and red color line for 30 N load).

Following the micro-scratch testing, the wear tracks of plain PLA and PLA-5GF were examined using a 3D surface profilometer integrated in the MFT-5000 platform (Rtec Instruments, San Jose, CA, USA) as shown in Figure 8. Under a nominal load of 15 N (Figure 8a,b), plain PLA exhibited a broad with pronounced pile-up, reflecting extensive plastic flow. In contrast, PLA-5GF showed a narrower, more defined track due to the constraint imposed by dispersed fibres, which limited chain mobility and localized deformation. At 30 N (Figure 8c,d), deformation increased in both systems, yet differences persisted: plain PLA revealed a wider groove with micro-tearing and delamination, whereas PLA-5GF maintained a shallower, more stable track. Quantitative profilometry (Figure 8e,f) confirmed these trends, with plain PLA showing a depth of ~69.7 µm and width of ~626.3 µm, compared to ~60.2 µm and ~481.5 µm for PLA-5GF. These results highlight that GF reinforcement enhances resistance to scratch-induced deformation by improving load distribution and suppressing localized plastic flow.

**Figure 9:**
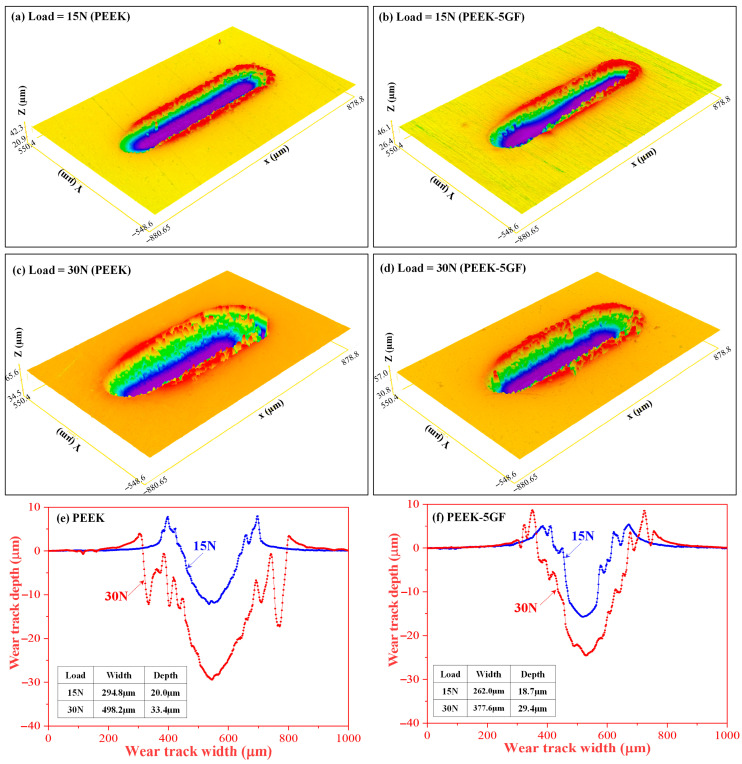
In situ OM images illustrating the micro-scratch morphologies of PEEK-based composites: (**a**,**c**) plain PEEK and (**b**,**d**) PEEK-5GF, subjected to scratching under varying loads. Images (**a**,**b**) correspond to a load of 15 N, while (**c**,**d**) present the morphologies under a higher load of 30 N. The corresponding surface profiles are shown in (**e**,**f**), providing insight into the depth and deformation characteristics associated with each loading condition (blue color line for 15 N and red color line for 30 N load).

A similar trend was observed for PEEK-based composites (Figure 9). At 15 N, plain PEEK (Figure 9a) exhibited deeper and broader grooves, reflecting ductile deformation and material removal, whereas PEEK-5GF (Figure 9b) showed shallower, narrower tracks due to fibre-induced constraint on polymer flow. At 30 N (Figure 9c,d), both systems displayed more severe damage; plain PEEK showed pronounced ploughing and pile-up, while PEEK-5GF exhibited matrix smearing, partial fibre exposure, and interfacial debonding. Surface profiles (Figure 9e,f) confirmed increased depth and width with load, yet GF reinforcement consistently reduced penetration and deformation. These results highlight the dual role of GF in enhancing load resistance while making performance increasingly dependent on fibre–matrix interfacial integrity under high stress.

**Figure 10:**
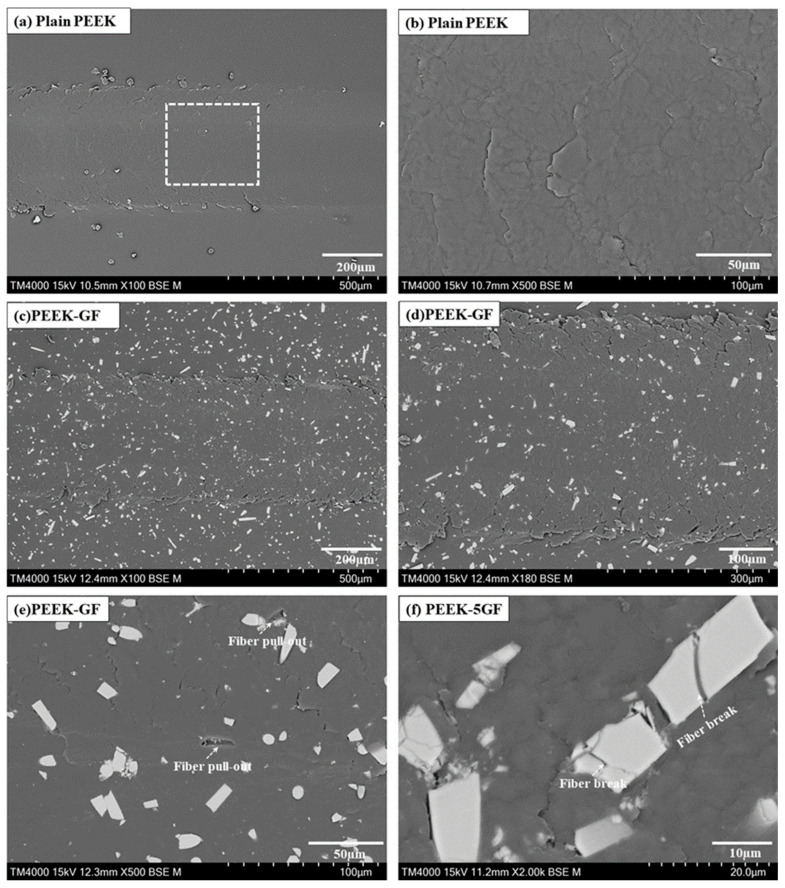
SEM micrographs illustrating the surface morphology of microscratch tracks generated under a nominal normal load of 30 N: (**a**,**b**) plain PEEK (the enlarged image Figure 10b taken from square marked zone in Figure 10a) and (**c**–**f**) PEEK-5GF.

A comprehensive investigation is undertaken on PEEK-based materials to elucidate the fundamental wear mechanisms activated during microscratching under a load of 30 N. Figure 10 presents SEM micrographs showing the surface morphology of the resulting scratch tracks. Figure 10a,b correspond to the plain PEEK specimen, whereas Figure 10c–f illustrate the microscratch morphology of the PEEK-5GF composite. The scratch tracks on the plain PEEK surface exhibited relatively uniform and continuous material flow, accompanied by pronounced plastic flow. Such morphological features are indicative of a predominantly ductile deformation mechanism with minimal microstructural disruption, consistent with the inherent homogeneity and viscoplastic behavior of the thermoplastic matrix.

By contrast, the PEEK-5GF specimens demonstrated distinctly different surface characteristics. The scratch tracks revealed clear evidence of fibre-mediated damage mechanisms, including fibre pull-out and fracture, as obvious in Figure 10c–f. The presence of partially embedded and protruding fibre fragments, alongside voids resulting from fibre detachment, as marked in Figure 10e, suggested interfacial debonding and insufficient load transfer across the fibre–matrix interface during tribological contact. Moreover, the alignment of broken fibre fragments along the scratch direction, as observed in Figure 10f, further supports the occurrence of localized brittle fracture within the reinforcing phase under high contact stresses. These findings collectively underscore the critical role of reinforcing fibres in modulating the deformation response and damage evolution of the composite during tribological loading. The transition from homogeneous plastic flow in the plain PEEK to a more intricate failure mechanism in the fibre-reinforced counterpart, characterized by matrix deformation, interfacial decohesion, and fibre fracture, highlights the complex interplay of constituent phases governing the scratch-induced wear behavior.

## 4. Conclusions

This investigation has undertaken a comprehensive examination of the fabrication methodologies, mechanical properties and tribological characteristics of PLA- and PEEK-based composites fabricated by additive manufacturing, with particular emphasis placed on elucidating the influence of GF reinforcement on the structural and functional attributes of the polymer matrices. The key findings derived from the experimental analyses are summarized as follows:

(i) Microstructural characterization revealed that the incorporated GF uniformly dispersed throughout both the PLA and PEEK matrices. No significant agglomeration, fibre pull-out, or interfacial debonding was observed, indicating strong interfacial adhesion between the GF and polymer phases. Such homogeneous dispersion is fundamental for enhancing the overall structural integrity and mechanical performance of the composites by facilitating efficient load transfer and minimizing stress concentrations within the polymeric network.
(ii) The mechanical performance evaluation demonstrated that the incorporation of GF reinforcement substantially enhanced the properties of both PLA- and PEEK-based composites compared to their plain matrices. Microhardness measurements revealed increases of 38.3% in the PLA composite and 36.3% in the PEEK composite. Similarly, the ultimate tensile strength of the PLA-based composite increased by 25.1%, while the PEEK-based composite exhibited a 13.4% improvement relative to their plain counterparts. These enhancements are primarily attributed to the effective stress transfer enabled by the strong interfacial adhesion between the GF and polymer phases, coupled with uniform fibre dispersion.
(iii) Tribological assessments revealed a significant enhancement in the wear resistance of the GF-reinforced polymer composites relative to the plain PLA and PEEK matrices. This improvement is attributed to the reinforcing effect of the GF, which bears a greater proportion of the applied load during micro-scratching and hinders the propagation of wear-induced damage. Furthermore, the strong interfacial bonding between the GF and polymer phases contributes to maintaining the composite’s structural integrity under tribological stress, thereby mitigating surface degradation and prolonging service life. These findings underscore the critical role of GF reinforcement in improving the durability and functional performance of additively manufactured polymer composites under frictional conditions.

## Data Availability

The original contributions presented in this study are included in the article. Further inquiries can be directed to the corresponding author.
